# Serotonin transporter antagonists target tumor-initiating cells in a transgenic mouse model of breast cancer

**DOI:** 10.18632/oncotarget.10614

**Published:** 2016-07-15

**Authors:** Robin M. Hallett, Adele Girgis-Gabardo, William D. Gwynne, Andrew O. Giacomelli, Jennifer N.P. Bisson, Jeremy E. Jensen, Anna Dvorkin-Gheva, John A. Hassell

**Affiliations:** ^1^ Department of Biochemistry and Biomedical Sciences, McMaster University, Hamilton, ON L8S 4K1, Canada; ^2^ Department of Pathology and Molecular Medicine, McMaster University, Hamilton, ON L8S 4K1, Canada; ^3^ Department of Biology, McMaster University, Hamilton, ON, L8S 4K1, Canada

**Keywords:** breast cancer, tumor-initiating cells, serotonin antagonists, anticancer stem cell drugs

## Abstract

Accumulating data suggests that the initiation and progression of human breast tumors is fueled by a rare subpopulation of tumor cells, termed breast tumor-initiating cells (BTIC), which resist radiotherapy and chemotherapy. Consequently, therapies that abrogate BTIC activity are needed to achieve durable cures for breast cancer patients. To identify such therapies we used a sensitive assay to complete a high-throughput screen of small molecules, including approved drugs, with BTIC-rich mouse mammary tumor cell populations. We found that inhibitors of the serotonin reuptake transporter (SERT) and serotonin receptors, which include approved drugs used to treat mood disorders, were potent inhibitors of mouse BTIC activity as determined by functional sphere-forming assays and the initiation of tumor formation by transplant of drug-exposed tumor cells into syngeneic mice. Moreover, sertraline (Zoloft), a selective serotonin reuptake inhibitor (SSRI), synergized with docetaxel (Taxotere) to shrink mouse breast tumors *in vivo*. Hence drugs targeting the serotonergic system might be repurposed to treat breast cancer patients to afford more durable breast cancer remissions.

## INTRODUCTION

Breast cancer was the first malignancy of solid epithelial tumors reported to follow the cancer stem cell (CSC) model [1]. Al-Hajj demonstrated that antibodies to the CD44 and CD24 cell surface antigens in combination with fluorescence-activated cell sorting (FACS) separated breast tumor cells into BTIC-enriched (CD44^+^/CD24^−^) and BTIC-depleted fractions as assessed by tumor cell transplantation into immune-compromised mice [1]. Limiting dilution tumor cell transplantation assays demonstrated that the bulk tumor cell population comprised ~0.01% BTIC, whereas the CD44^+^/CD24^−^ fraction constituted ~1–2% BTIC. Subsequent analyses of mammary tumors from various transgenic mouse models of breast cancer revealed that the majority of these also follow the CSC model [2–6].

The CSC model proposes that genomic alterations in tissue-specific cells results in clonal tumor cell populations with stem cell-like properties, including the capacity for limitless self-renewal and differentiation [7]. Thus tumors following the CSC model comprise a cellular hierarchy of BTIC at their apex and non-tumorigenic differentiating BTIC progeny at their base. Recent findings demonstrate that the induction of an epithelial to mesenchymal transition can endow “non-tumorigenic” tumor cells with BTIC activity implying that tumor cells may transition between non-tumorigenic and tumorigenic states [8–10]. Hence the bulk non-tumorigenic tumor cells may provide a reservoir of BTIC. These observations have therapeutic implications [11–13]. Conventional cytotoxic therapies principally eradicate the non-tumorigenic progeny of BTIC, which comprise the vast majority of cells populating tumors. Consequently tumors regress after radiotherapy or chemotherapy, but often recur likely due to therapy-resistant BTIC. Thus to ensure durable breast cancer remissions anticancer therapies should eradicate BTIC as well as their non-tumorigenic progeny.

Identifying molecular targets required for maintaining BTIC activity would provide an avenue to develop anti-BTIC therapies. However, discovering such targets has been difficult to achieve due to the scarcity of BTICs in human breast tumors [14] or breast tumor cell lines [15]. We recently reported that various transgenic mouse models of breast cancer, including those shown to follow the CSC model, comprise a high fraction of BTIC [6], as determined by limiting dilution tumor cell transplantation experiments, the “gold standard” assay for tumor-initiating cells [14]. BTIC frequencies in the tumors of 3 different models averaged 30%; indeed, single tumor cells induced tumors at high frequency [6]. Moreover, we also showed that tumor cells isolated from mouse mammary tumors maintain a high BTIC frequency [6] when propagated in serum-free, chemically-defined medium first developed to culture mouse neuronal stem and progenitor cells [16]. By contrast, propagating the tumor cells in serum-containing medium reduced BTIC frequencies by 4–5 orders of magnitude [6]. We reasoned that these models would enable us to complete high-throughput screens with BTIC-enriched mouse mammary tumor cells and thereby identify agents targeting BTIC, which could be subsequently tested for their capacity to abrogate the tumorigenicity of their human counterparts. Here we report that SSRI, antagonists of the serotonin (5-hydroxytryptamine; 5-HT) transporter, targeted BTIC in tumors of the MMTV-Neu (N202) model [17] of breast cancer. Interestingly, 5-HT signalling has previously been implicated in postnatal mammary gland development [18, 19] and linked to breast cancer [20].

## RESULTS

### Serotonergic system antagonists compromise mouse mammary tumor cell viability

We previously reported that free-floating sphere cultures of MMTV-Neu derived tumor cells [17], which we termed tumorspheres [21], could be readily and reproducibly established from primary tumor cells and serially propagated *in vitro* [6]. The BTIC frequency of tumorsphere-derived cells is about half that of primary tumor cells and averages 15%. We speculated that this high BTIC frequency might make it be possible to identify compounds targeting BTIC.

**Figure 1:**
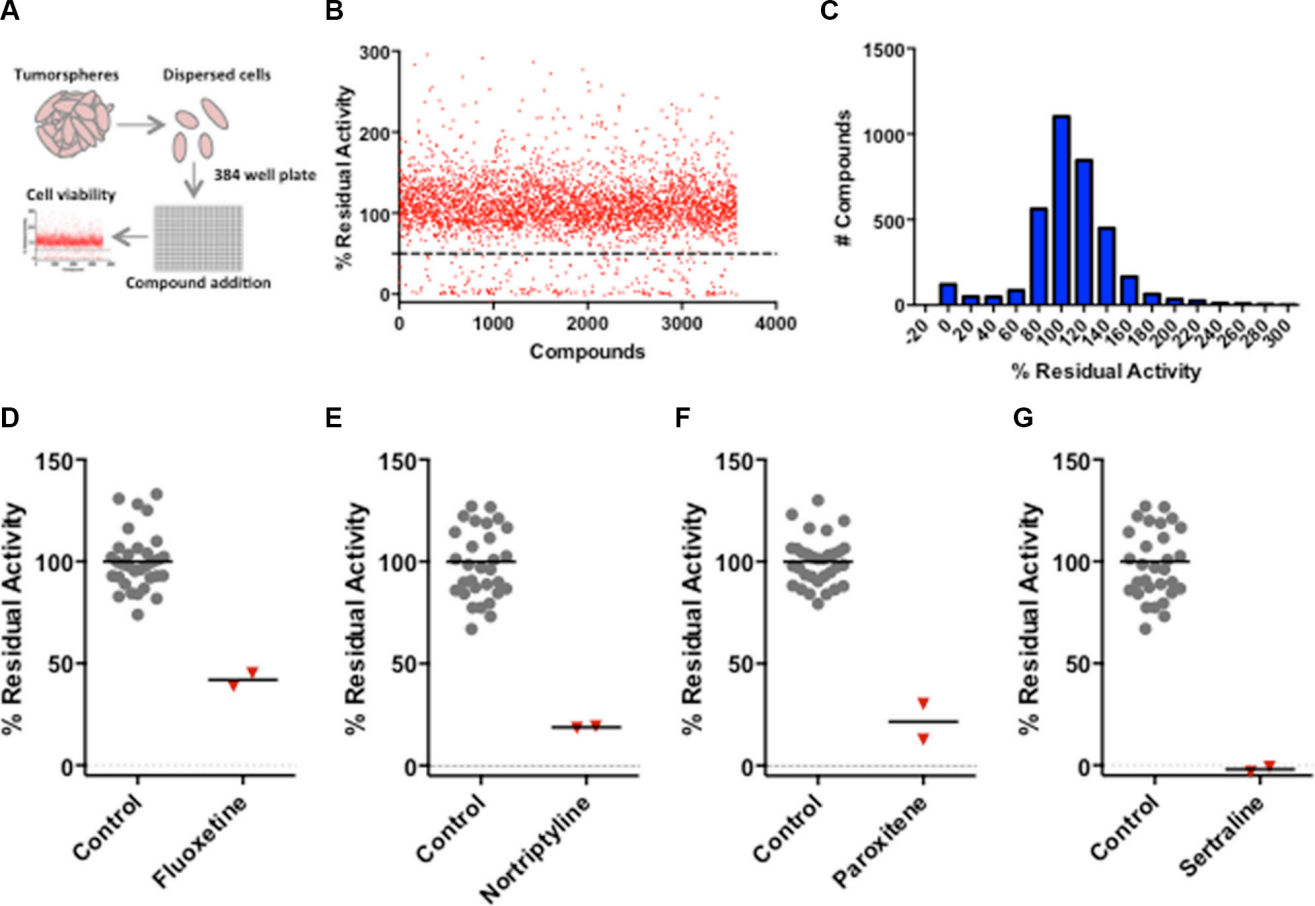
High-throughput screening of BTIC-enriched mouse derived breast tumor cells identifies 5-HT antagonists as potential breast cancer therapeutics (**A**) Schematic depicting the experimental pipeline used to identify candidate compounds affecting tumor cell viability. (**B**) Scatter plot showing the screening results of the bioactive subset of the Canadian Compound Collection. (**C**) The histogram illustrates that the compound activities approximately conform to a Gaussian distribution. (**D**-**G**) The primary screening data illustrating the effect of the antagonists (fluoxetine, nortriptyline, paroxetine and sertraline) at a concentration of 5 μM in duplicate wells of 384-well plates. The positive control values represent the multiple tumor cell samples that were exposed to the vehicle.

We used a sensitive alamarBlue assay [22] to perform a high-throughput screen to identify small molecules that reduced tumorsphere-resident cell viability (Figure 1A). In short, freshly dissociated tumorsphere-derived cells were seeded into 384-well plates with test compounds (5 micromolar [μM] in duplicate wells), placed in chemically-defined medium conducive for sphere formation for 48 hours, and assayed for their capacity to reduce alamarBlue, a measure of the reducing environment in cells, which indirectly reflects cell viability. The chemical library comprised roughly 35,000 small molecules including a subset of approximately 3,500 bioactive compounds and drugs.

A scatter plot (Figure 1B) and histogram (Figure 1C) of the alamarBlue residual activity data for the bioactive small molecules illustrated that their activities were normally distributed with mean ~100% residual activity and a standard deviation of 37%. Compounds that reduced tumor cell viability by greater than 50% were considered hits; independently sourced fresh compounds were selected for verification at a range of compound concentrations thus establishing their half-maximal inhibitory concentration (IC_50_). The verified hits included SSRI (fluoxetine, paroxetine and sertraline) and both non-selective and selective antagonists of one or more 5-HT receptors, which are encoded by a multi-gene family comprising 14 genes in mice. The inhibitory activity of the duplicate samples of the SSRI and a non-selective antagonist (nortriptyline) from the primary screen compared to that of vehicle (DMSO) controls is illustrated in Figure 1 panels D–G. We focussed our analyses on the SSRI because they are highly selective drugs with an established safety profile and are widely used for sustained periods to primarily treat depression.

### Expression of SERT, TPH1 and serotonin in mouse breast tumors

**Figure 2:**
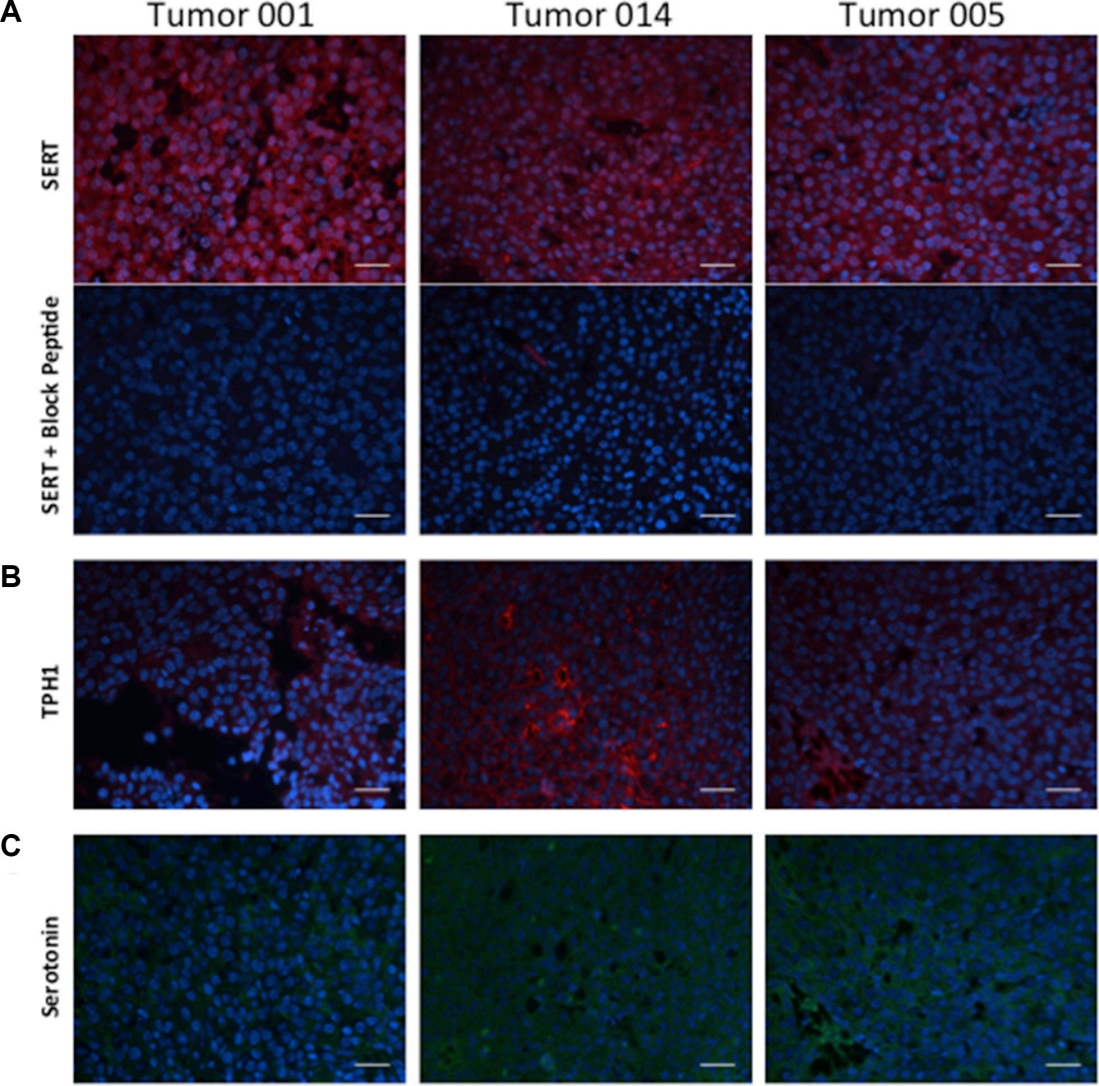
Expression of SERT, TPH1 and 5-HT in 3 independent tumors from the MMTV-Neu transgenic strain (**A**) Independent tumor sections were incubated with a polyclonal antibody to SERT without or with a blocking peptide, the antigen used to elicit antibody production in rabbits. (**B**) Independent tumor sections were incubated with an antibody to TPH1. (**C**) Independent tumor sections stained with an antibody that specifically binds to 5-HT. Primary antibodies to SERT (red), TPH1 (red) and 5-HT (green) were used in combination with fluor-labeled secondary antibodies as described in Materials and Methods. The scale bar represents 50 micrometers (μm).

To determine whether SERT, the molecular target of the SSRI, was indeed expressed in mammary tumors, we prepared sections from 3 independent tumors and exposed them to a SERT-specific polyclonal antibody. Analyses of the sections revealed that SERT was expressed in most of the tumor cells from each of the 3 tumors examined (Figure 2A, upper panels). Incubation of the tumor sections with a SERT blocking peptide (the antigen used to derive the antibody) completely abrogated binding by the SERT antibody (Figure 2A, lower panels).

To identify the cellular source of 5-HT in mouse mammary tumors we stained the same tumor sections with an antibody to TPH1, the rate-limiting enzyme required for 5-HT biosynthesis from tryptophan in non-neuronal tissues. Like SERT, TPH1 was also expressed in the majority of the tumor cells in the 3 mouse mammary tumor sections we examined (Figure 2B). To determine whether TPH1 is active in the tumor cells we enquired whether 5-HT is present in the tumor cells using an antibody specific to 5-HT. We found that 5-HT was present in most of the tumor cells comprising the tumor sections suggesting that TPH1 is indeed active in the tumor cells (Figure 2C). Taken together, these data suggest that mouse mammary tumor cells possess the machinery to synthesize and transport 5-HT implying a functional role for 5-HT in breast tumor cells.

### Serotonergic system antagonists target sphere-forming cells

To determine whether compounds selective for serotonergic pathway components target BTIC we initially assessed their capacity to affect sphere formation, a commonly used albeit controversial [23], functional *in vitro* surrogate assay for both mammary epithelial stem cells (MESC) and BTIC [5, 8, 24–27]. Various studies have shown that MESC [25] and BTIC co-fractionate with sphere-forming cells after FACS [15, 28], and that agents that alter MESC/BTIC frequency similarly affect the frequency of sphere forming cells [6, 29, 30] suggesting that MESC/BTIC are endowed with sphere forming capacity.

We have shown that spheres arise from dispersed tumorsphere-derived cells in direct proportion to the number of cells plated into the medium, and that the frequency of sphere-forming cells, which averages 1% of the total tumor cell population, can be accurately quantified over a range of cell densities [29]. Moreover, plating single tumorsphere-derived cells into the wells of 96-well plates yielded spheres at the same frequency (1–3%) as that arising when the cells are plated at higher cell densities (Supplementary Figure 1).

**Figure 3:**
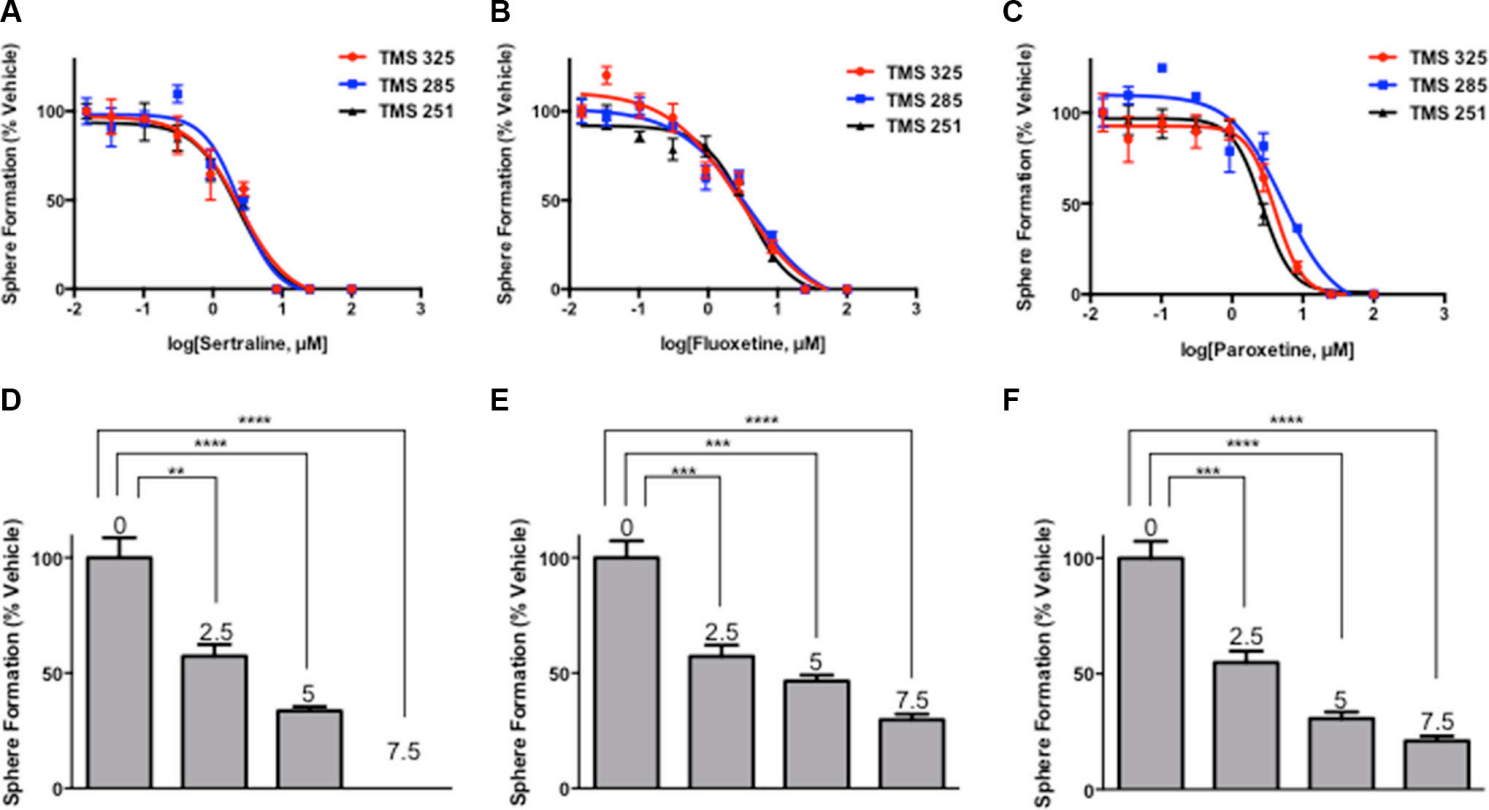
SSRIs irreversibly target tumorsphere-forming cells (**A**–**C**) Sertraline, fluoxetine and paroxetine inhibit sphere formation by tumorsphere-derived cells prepared from 3 independent MMTV-Neu tumors. (**D**–**F**) Secondary sphere forming assays reveal that exposure of tumorsphere-derived cells to sertraline, fluoxetine and paroxetine during primary sphere-forming assays irreversibly reduced the frequency of sphere-forming cells in SSRI-free medium in a dose-dependent manner. The numbers above the bars indicate the concentration of each SSRI used in the primary sphere-forming assay. One-way ANOVA *P* < 0.0001 (panels D–F).

Each of the highly selective SSRIs tested (sertraline, fluoxetine [Prozac] and paroxetine [Paxil]) reduced sphere formation by tumorsphere-derived cells in a dose-dependent fashion in each of 3 tumorsphere cultures established from independent tumors arising in different transgenic mice compared to those that were exposed to the vehicle (DMSO) (Figure 3A–3C). Sertraline inhibited sphere formation with an IC_50_ of between 2.3–2.5 μM in the 3 tumorsphere cultures, and completely abrogated sphere formation at approximately 8–9 μM. Similarly, fluoxetine and paroxetine inhibited sphere formation in each of the tumorsphere preparations. The IC_50_ values of fluoxetine varied between 2.8–3.9 μM, whereas those for paroxetine fluctuated between 2.7–5.3 μM among the tumorsphere preparations. Hence all 3 SSRIs targeted the sphere-forming cells established from multiple independent mouse mammary tumors with similar IC_50_ values.

We have shown that agents that irreversibly affect sphere formation induce apoptotic and differentiation programs, largely irreversible biological processes, which require a sustained period for their execution and which reduce BTIC frequency [29, 31]. To learn whether the SSRIs acted by a reversible or irreversible mechanism the spheres that arose after exposure to the SSRIs at select concentrations in primary sphere-forming assays were dissociated, the same number of dispersed viable cells from each sample were seeded into SSRI-free medium for another 96 hours, and the number of spheres that arose in the secondary sphere-forming assays was determined and compared to those arising after exposure of the tumor cells to the vehicle.

The tumorsphere-derived cells exposed to the vehicle formed spheres in the secondary sphere-forming assay at the same frequency (~1%) as they did in the primary sphere-forming assay (Figure 3, compare panels 3A and 3D, 3B and 3E, and 3C and 3F). By contrast, exposure of the tumorsphere-derived cells to the SSRI during the primary sphere-forming assay reduced their capacity to subsequently form new spheres in a dose-dependent fashion at each concentration of the SSRI tested. For example, tumorsphere-derived cells treated with the approximate IC_50_ dose of sertraline (2.5 μM) exhibited a 50% reduction in their capacity to form spheres in the secondary sphere-forming assay even though sertraline was not present during secondary sphere formation. The use of 7.5 μM sertraline, the approximate IC_90_ dose for sphere formation in the primary sphere-forming assay, completely abrogated sphere formation in the secondary sphere-forming assay suggesting that this dose completely eradicated sphere-forming cells. Fluoxetine and paroxetine similarly irreversibly inhibited secondary sphere formation. Hence, 4-day exposure of tumorsphere-derived cells to SSRI was sufficient to irreversibly inhibit their capacity to subsequently form new spheres in the absence of the SSRI illustrating that the SSRI targeted the sphere-forming cell subpopulation of tumorpheres.

The occurrence of TPH1 in the mammary tumor cells prompted us to learn whether the enzyme was functionally required for sphere formation. We assessed the effect of a TPH1 inhibitor (LP533401) [32] on sphere formation by tumorsphere-derived cells. We used tumorspheres prepared from 3 independent mammary tumors different from those used above to complete quantitative sphere forming assays. LP533401 inhibited sphere formation with an IC_50_ between 6.8–8.6 μM among the 3 independent tumorsphere cultures (Supplementary Figure 2).

Two of the hits from our screen targeting 5-HT receptors are selective for different receptors implying that inhibition of the activity of any one of them reduced sphere formation. Hence we assessed the activity of additional selective inhibitors including those identified in the screen in sphere-forming assays. Among the antagonists tested those selectively targeting 5-HT_1B_, 5-HT_2C_, 5-HT_5A_ and 5-HT_6_ all inhibited sphere formation (Supplementary Table 1). Assuming that the reported selectivity of the receptor antagonists is manifest at the IC_50_ values in the sphere-forming assays, these findings imply that the activity of as many as 4 receptors is required for this process.

Collectively our data demonstrates that structurally diverse compounds targeting TPH1, SERT and multiple 5-HT receptors all inhibited sphere formation consistent with the notion that the effect of the antagonists resulted from inhibition of the activity of their molecular targets and was not due to off-target effects.

### Sertraline targets BTIC

Our data showing that SSRIs irreversibly inhibited sphere formation by tumorsphere-derived cells suggested that the SSRI targeted BTIC. To directly test this possibility we incubated freshly dissociated tumorsphere-derived cells into medium with the vehicle or with sertraline at either 5 μM or 7.5 μM. Following 96 hours of exposure to the vehicle or sertraline, any spheres that formed were collected, dissociated and equal numbers of viable cells were transplanted into one of the #2 fat pads of 10 6–8 week old syngeneic female mice (FVB/N strain). We monitored tumor incidence and volume over a 15-week period, which represented approximately 4 weeks after all 10 mice transplanted with vehicle-treated tumor cells had developed palpable tumors.

**Figure 4:**
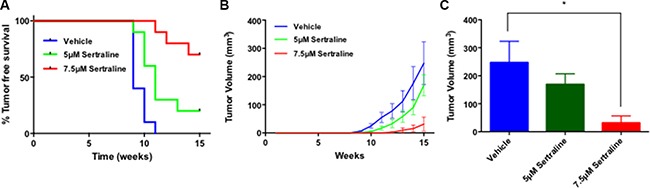
Sertraline targets BTIC *ex vivo* (**A**) Mouse mammary tumor derived cells exposed to sertraline *in vitro* under sphere-forming conditions yield tumors after transplant into syngeneic mice with reduced frequency and increased latency compared to vehicle-treated cells. The Kaplan-Meier curve comparisons were performed using the log-rank test; *P* = 0.0003. (**B**) Sertraline treated tumor cells formed tumors that grow more slowly *in vivo* after transplant than did those exposed to the vehicle. One-way ANOVA *P* = 0.02. (**C**) After 15 weeks tumors formed by sertraline-treated tumor cells (7.5 μM) are on average substantially smaller than those formed by vehicle treated tumor cells. *P*-value (*) < 0.05.

Tumors were detected in 6 of the 10 mice transplanted with vehicle-treated tumor cells at week 9 following tumor cell transplantation, whereas only 1 of the 10 mice transplanted with tumor cells exposed to 5 μM sertraline developed tumors and none of the mice transplanted with tumor cells exposed to 7.5 μM sertraline developed tumors (Figure 4A). Every mouse transplanted with vehicle-treated cells developed a palpable tumor by 11-weeks post transplant, whereas at 11 weeks only 7 of 10 mice transplanted with tumor cells exposed to 5 μM sertraline developed tumors, and only 1 of the 10 mice transplanted with tumor cells treated with 7.5 μM sertraline developed tumors. Furthermore, at 15 weeks post transplant, 8 of 10 mice that had been transplanted with tumor cells exposed to 5 μM sertraline developed tumors, but only 3 of 10 mice transplanted with tumor cells exposed to 7.5 μM sertraline developed tumors. In line with these findings average tumor volume during the 15-week period after transplant of the tumor cells was greatest in the mice transplanted with vehicle-treated tumor cells compared with those arising in mice transplanted with sertraline-treated tumor cells (Figure 4B). By 15 weeks post transplant the average tumor volumes in mice transplanted with the vehicle-treated tumor cells were 10-fold greater than those of mice that had been transplanted with tumor cells exposed to 7.5 μM sertraline (Figure 4C).

We have previously reported that the time to appearance and volume of tumors arising after primary tumor cell or tumorsphere-derived cell transplant into syngeneic mice is directly proportional to the frequency of BTIC in the transplanted tumor cell population using the same transgenic model employed in these studies [6]. Moreover, observations similar to ours were reported for human melanoma tumor cells transplanted into immune-compromised mice [33]. Hence the data reported above suggests that sertraline targets BTIC.

### Sertraline synergizes with docetaxel to inhibit sphere formation

Any clinical studies to assess the activity of sertraline as an anticancer agent will likely be carried out by treating terminal breast cancer patients with both sertraline and chemotherapy or radiotherapy. We also imagine that achieving durable breast cancer remissions in patients will require targeting both the infrequent BTIC tumor cell subpopulation and the abundant non-tumorigenic tumor cell subpopulation, a potential source of BTIC. Hence we sought to learn whether sertraline and docetaxel, a chemotherapeutic often used to treat breast cancer patients, might be combined to perturb tumor growth in mice.

We initially determined what concentrations of sertraline and docetaxel could be combined to affect tumor cell viability *in vitro* using alamarBlue assays. To this end we seeded freshly dissociated tumorsphere-derived cells into the wells of a 384-well plate and added sertraline and/or docetaxel in a dose matrix that included 8 concentrations of only sertraline or docetaxel, and all possible dose combinations of both drugs in quadruplicate. After 48 hours in culture, we assessed the viability of the cells in each well using alamarBlue, and compared the measured with the predicted alamarBlue residual activity for all dose combinations following the Bliss model of additivity [34]. Similar measured and predicted residual activities would indicate an additive effect of sertraline and docetaxel, whereas less than additive measured values would indicate a masking effect, and greater than additive measured values would imply that the drugs functioned synergistically.

**Figure 5:**
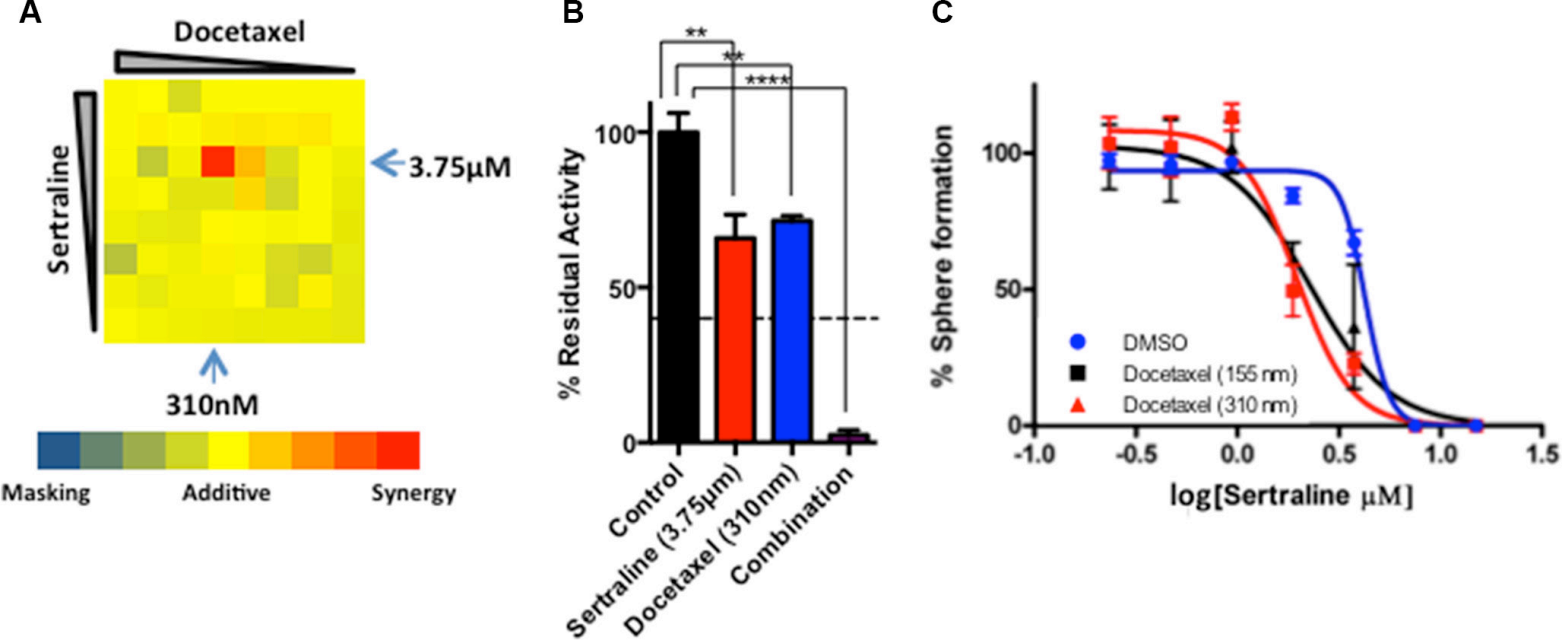
Docetaxel and sertraline act synergistically to target tumorsphere-forming cells *in vitro* (**A**) Dose combination matrix for docetaxel and sertraline illustrating their effect on alamarBlue reduction. (**B**) Bar diagram demonstrating the synergistic effect of sertraline and docetaxel at the IC_50_ for each drug in alamarBlue assays. The dashed line depicts the expected extent of inhibition of alamarBlue reduction if the effect of the drugs was additive. One-way ANOVA *P* < 0.001. (**C**) The effect of docetaxel (at two concentrations) and sertraline (at multiple concentrations) in combination on sphere formation.

At most concentrations of each drug, the interaction between sertraline and docetaxel was additive; however, we observed substantial synergy in and around the IC_50_ for sertraline (3.75 μM) and that for docetaxel (310 nanomolar; nM) (Figure 5A, indicated by blue arrows). Whereas both sertraline and docetaxel individually reduced tumor cell viability by approximately 30–40%, their combination at these concentrations resulted in a near 100% reduction in cell viability (Figure 5B; dotted line indicates predicted additive relationship).

We followed up these experiments with sphere-forming assays using a range of sertraline concentrations in the absence or presence of docetaxel at either 155 nM or 310 nM. We seeded dispersed tumorsphere-derived cells into medium containing various concentrations of sertraline without and with each of the two concentrations of docetaxel, and counted spheres after 96 hours (Figure 5C). As observed previously, sertraline inhibited sphere formation with an IC_50_ of approximately 3.5 μM (Figure 5C, blue line). Each concentration of docetaxel decreased the IC_50_ of sertraline by 2–3-fold (Figure 5C, red and black lines). Hence sertraline and docetaxel synergized to inhibit mammary tumor cell viability and their capacity to form spheres *in vitro*.

### Sertraline synergizes with docetaxel to shrink mammary tumors

**Figure 6:**
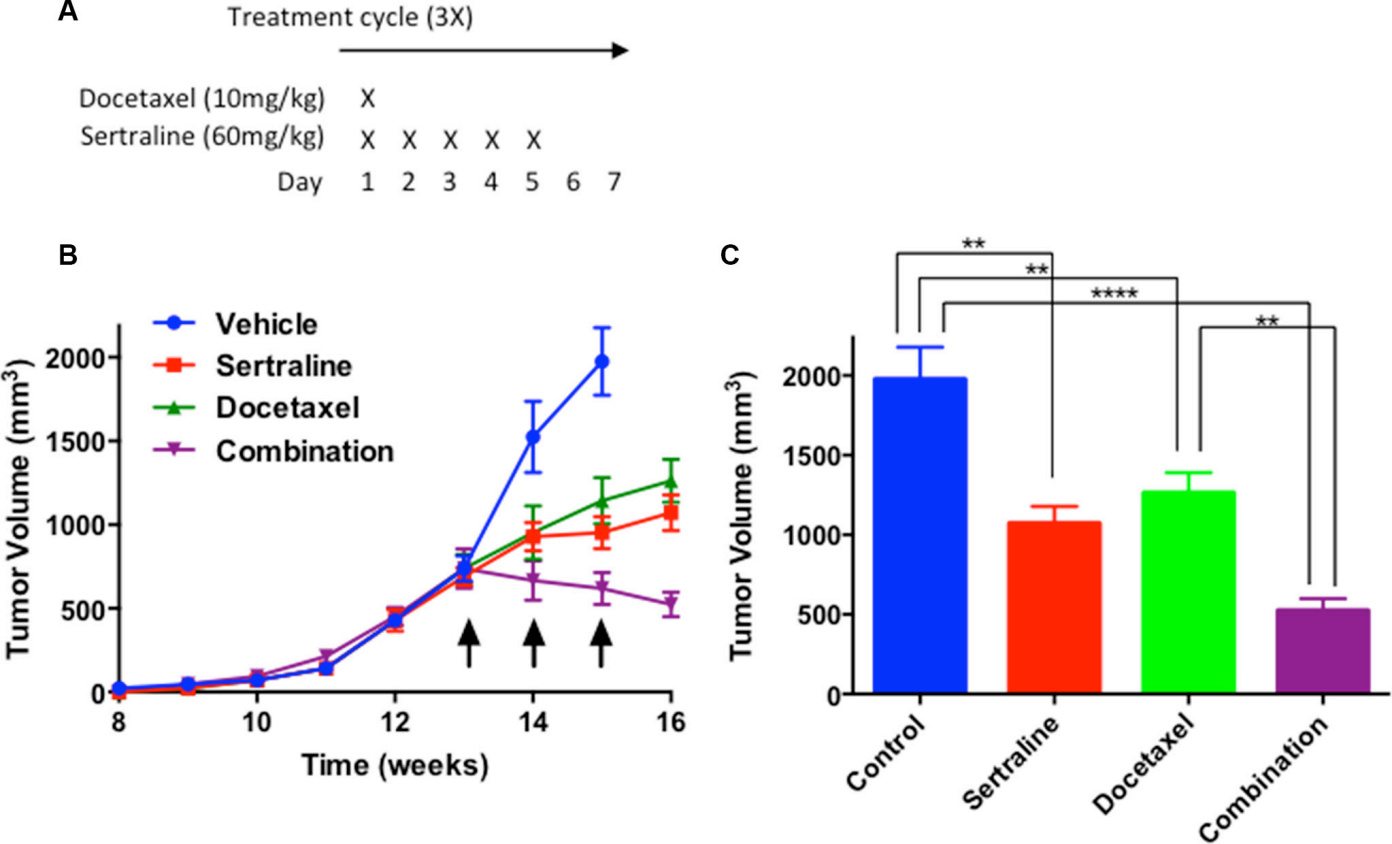
In combination sertraline and docetaxel inhibit tumor growth to a greater extent than either drug individually (**A**) Docetaxel and sertraline were administered on the first day of the treatment regimen, whereas thereafter sertraline was administered daily for another 4 consecutive days. No treatments were provided for 2 consecutive days after the 5-day treatment period. (**B**) Sertraline or docetaxel modestly inhibited tumor growth rate, but the combination of both drugs shrank tumors. (**C**) The combination of sertraline and docetaxel reduced final tumor volume to a much greater extent than did either drug individually. On-way ANOVA *P* < 0.0001.

We next sought to determine whether sertraline alone or in combination with docetaxel affected the growth of established mammary tumors. In short, we orthotopically transplanted freshly isolated tumor cells from tumors of the MMTV-Neu (N202) transgenic strain into syngeneic female mice to elicit tumor growth. When the tumors achieved an average volume of approximately 700 mm^3^ - 13 weeks after transplant of the primary tumor cells - we treated the tumor-bearing mice with the vehicle, sertraline, docetaxel or a combination of sertraline and docetaxel using the schedule outlined in Figure 6A. Treatment of mice with the drugs occurred over the course of 3 weeks; the mice were sacrificed a week after the third treatment cycle (Figure 6B). Because mice that were administered the vehicle reached end point (~2,000 mm^3^, equivalent to 10% of their body mass) after the second treatment cycle they were sacrificed 15 weeks after injecting the tumor cells.

A week after initiating treatment, the tumors of the vehicle-treated mice increased in volume (Figure 6B, blue line), and achieved a final average volume of ~2,000 mm^3^ after another week (Figure 6C, blue box). The tumors of mice individually administered sertraline or docetaxel also increased in volume after treatment started, but to a lesser extent than those of mice treated with the vehicle (Figure 6B, compare blue line to the red and green lines). The growth inhibitory effect of the individual drugs was manifest a week after the first treatment occurred. A week after the third and last drug treatment cycle the tumors of the mice administered each drug individually continued to increase but were significantly smaller than those of the mice administered the vehicle (Figure 6C). By contrast, the volume of the tumors of mice treated with both sertraline and docetaxel decreased after the first treatment cycle and continued to decline after the third treatment cycle ended (Figure 6B, purple line). The final tumor volumes at end point (15 weeks) for the mice administered the vehicle (control), and those of mice administered sertraline, docetaxel or the combination of both drugs at the end of 16 weeks were 52%, 64% and 26% the volume of the controls respectively (Figure 6C). Because the vehicle-treated mice were sacrificed a week earlier than those treated with the individual drugs or their combination, the growth inhibitory effect of the drugs would have been greater had all the mice been sacrificed at the same endpoint. Importantly the combination of both drugs reduced tumor growth rate and volume to a greater extent than did each drug individually.

**Figure 7:**
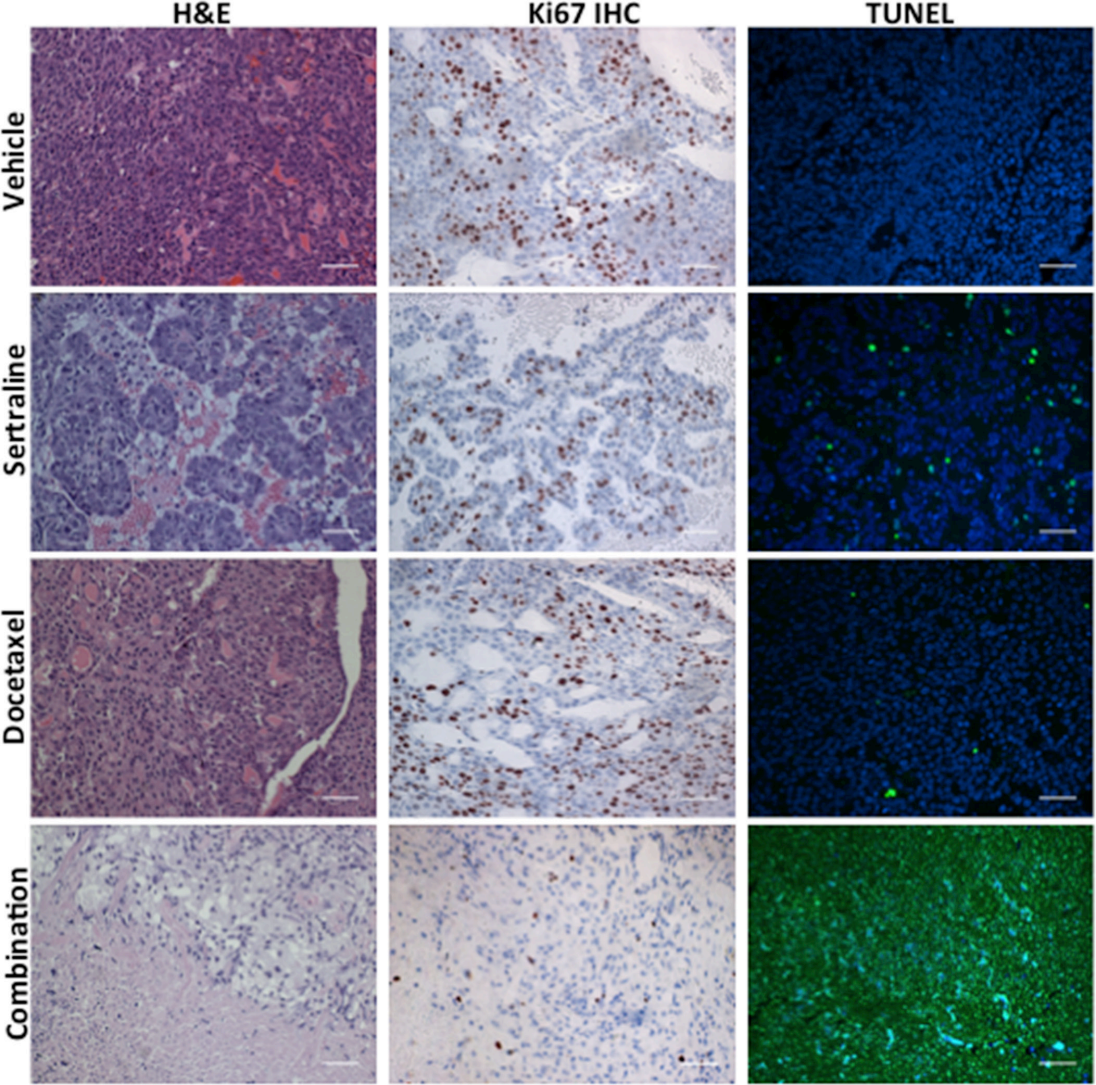
The combination of sertraline and docetaxel reduces the frequency of proliferating cells and increases that of apoptotic cells to a greater extent than either drug alone Tumors from mice administered the vehicle, sertraline, docetaxel, or the combination of each drug were sectioned and these were stained with H&E, antibodies to Ki67 or assayed for fragmented DNA using the TUNEL assay. The scale bars represent 50 μm.

To uncover potential mechanisms by which sertraline and/or docetaxel affected tumor growth, we prepared histological sections from tumors of each cohort and stained them with hematoxylin and eosin (H&E). Tumors harvested from mice treated with sertraline were morphologically distinct from those of mice administered the vehicle (Figure 7; compare panels of tumor sections stained with H&E from the vehicle- and sertraline-treated mice). The tumors of mice administered sertraline comprised clusters of tumor cells separated by cell-free areas, which generally contained red blood cells. Tumors harvested from docetaxel-treated mice appeared histologically similar to those of the vehicle-treated mice, this despite the tumors being on average 64% the volume of those of the vehicle-treated mice (Figure 7, panel labeled H&E and docetaxel). By contrast, tumors harvested from mice that were treated with the combination of sertraline and docetaxel were largely devoid of tumor cells, and comprised regions of what appeared to be stromal cells and cellular debris (Figure 7, panel labeled H&E and combination).

To learn what cellular processes were affected by sertraline, docetaxel or their combination we enquired whether their effect could be ascribed to changes in tumor cell proliferation or apoptosis. We used immunohistochemistry with antibodies to Ki67, a marker of cell proliferation, to estimate the frequency of proliferating cells in tumor sections from all 4 treatment-groups (Figure 7, Ki67 IHC). Sertraline reduced the frequency of Ki67-positive tumor cells by 55% (47% in the controls versus 26% in the sertraline treatment cohort) (Figure 7, Ki67 IHC and sertraline: Supplementary Figure 3). Docetaxel had little effect on the frequency of Ki67-positive cells in the tumor sections compared to those from the tumors of mice treated with the vehicle. However, we observed a significant decrease in Ki67 positive cells in tumor sections from mice treated with the combination of sertraline and docetaxel. There was a rough correspondence between the final volume of individual tumors in each treatment cohort at endpoint and the fraction of Ki67-positive cells.

We similarly completed TUNEL assays with tumor sections of all the treatment cohorts to determine whether the drugs affected tumor cell apoptosis (Figure 7; panels labelled TUNEL). Whereas we observed a modest increase in the frequency of TUNEL-positive cells in tumor sections from the sertraline- or docetaxel-treated mice compared to mice administered the vehicle, tumors of mice administered both drugs comprised a very high frequency of TUNEL-positive cells. As observed in the H&E stained tumor sections, the tumors of mice treated with both drugs comprised very few tumor cells, and in those rare areas comprising cells, all the cells were undergoing apoptosis.

Taken together these observations suggest that treatment of tumor-bearing mice with sertraline reduced tumor growth rate by inhibiting tumor cell proliferation and modestly increasing their apoptosis. Docetaxel also reduced the rate of tumor growth, but had little effect on the frequency of proliferating tumor cells or that of apoptotic cells. However, the combination of both drugs shrank tumors, and dramatically reduced their frequency of proliferating tumor cells and increased that of apoptotic cells.

To learn whether the drugs targeted tumor-resident BTIC we sought to determine their frequency in residual tumor masses remaining after drug treatment. As a rapid means to this end we performed sphere-forming assays reasoning that if a drug or drug combination targeted BTIC *in vivo*, cells isolated from residual tumor masses would comprise a reduced frequency of sphere-forming cells. To this end, we harvested tumor cells from residual tumors after drug treatment, and seeded equal numbers of viable tumor cells at limiting dilutions into serum-free, chemically-defined medium. Whereas tumor cells isolated from vehicle-, sertraline- and docetaxel-treated tumor-bearing mice all formed spheres with roughly similar frequencies, those viable cells isolated from residual tumor masses remaining after treatment with the combination of these drugs displayed an approximate 60% reduction in their sphere forming efficiency (Supplementary Figure 4). These data suggests that sertraline targeted BTIC *in vivo*.

## DISCUSSION

Whereas TIC have been found in human and mouse tumors of diverse tissue origin [6, 35–41], relatively few compounds have been identified that target these cells [29, 31, 42, 43]. Identifying agents targeting BTIC is an important objective as many reports suggest that these cells are resistant to conventional radio- and chemotherapies [38, 42, 44, 45]. Indeed, chemotherapy increases the fraction of BTIC in human breast tumors by selectively targeting the non-tumorigenic tumor cell population [46].

To identify BTIC targeting small molecules we employed BTIC-enriched tumor cells from a transgenic mouse model of breast cancer anticipating that among the mouse BTIC-targeting agents we identified would be those that also target human BTIC. We initially focussed our analyses on the hits among the ~3,500 bioactive compounds. Interestingly, the hits included 28 antagonists of neurotransmitter activity, which comprised the third largest class of such compounds after antibiotics (39 small molecules) and inhibitors of cell signalling (30 compounds). Collectively these 3 compound classes comprised roughly 50% of all the bioactive hits. Nine serotonergic antagonists targeting SERT or 5-HT receptors were identified in our screen.

All the serotonergic antagonists inhibited sphere formation by tumorphere-derived cells. Moreover, other selective antagonists targeting TPH1, SERT and up to 4 different 5-HT receptors reduced the frequency of tumorsphere-forming cells. These various observations suggest that breast tumor cells have the capacity to synthesize 5-HT, which acts through SERT and multiple 5-HT receptors to maintain tumorsphere-forming cell activity. The fact that structurally unrelated serotonergic antagonists affecting the activity of different molecular targets all reduced the frequency of tumorsphere-forming cells strongly implies that the serotonergic system is required for their functional activity. In this regard it is noteworthy that knockout of *Slc6a14*, which encodes a transporter for neutral amino acids including tryptophan, the precursor of 5-HT, compromises mammary tumorigenesis in mouse models of breast cancer [47]. Importantly, a recent analysis of a genome-wide lentivirus shRNA dropout screen in over 70 human breast tumor cell lines [48] provided the opportunity for us to determine whether shRNAs targeting serotonergic pathway component transcripts declined during passage of the cell lines *in vitro*. Indeed transcripts encoding TPH1, SERT and the 5-HT receptors declined with passage number in between 40%–90% of 72 breast tumor cell lines for which data was available at the time of plating the tumor cells, and after each of 2 passages. The loss of sequences encoding shRNAs targeting serotonergic pathway components occurred independent of the molecular subtype of the breast tumor cell lines. These functional genomic analyses strongly suggest that the serotonergic pathway components contribute to breast tumor cell survival/proliferation, and imply that the structurally diverse 5-HT antagonists targeting distinct serotonergic system components we used for our experiments likely did not result from off-target effects.

Whereas a functional role for 5-HT in mammary tumor cells was unexpected, studies pioneered by Horseman and his colleagues have implicated 5-HT in postnatal mouse and bovine mammary gland development [19, 49]. Initial studies to discover prolactin target genes in the mammary glands of mice identified *Tph1* [19]. TPH1 transcripts are increased during pregnancy and lactation leading to increased 5-HT levels in the mammary epithelium. 5-HT acts in a negative feedback loop to suppress prolactin stimulation of milk production during lactation and to initiate involution by inducing epithelial cell apoptosis [49]. The activity of 5-HT requires 5-HT receptor activity because a non-selective receptor antagonist, methysergide, inhibited the effect of 5-HT on the expression of milk proteins and on 5-HT mediated initiation of apoptosis [19]. 5-HT also increases the synthesis of parathyroid hormone-related peptide, which acts on bone to release calcium that ultimately accumulates in milk. Current data suggests that 5-HT binding to the 5-HT_7_receptor triggers mammary epithelial cell apoptosis during the involution phase of postnatal mammary gland development, whereas 5-HT binding to the 5-HT_2B_ receptor initiates the expression of parathyroid hormone-related peptide (PTHrP) from mammary epithelial cells during lactation [50–54]. Hence 5-HT acting through different receptors mediates distinct cellular processes during postnatal mammary gland development. By analogy, the apparent requirement for multiple different 5-HT receptors for BTIC activity may reflect the fact that each receptor acts by a different mechanism to sustain particular attributes of these tumorigenic cells.

The serotonergic system including TPH1, SERT and numerous 5-HT receptors are also expressed in human breast tumor cell lines [55, 56] and breast tumors [53, 57]. Interestingly TPH1 protein levels are increased in primary tumors that had metastasized to lymph nodes suggesting a relationship between tumor aggressiveness and increased 5-HT levels in tumor cells [55]. In keeping with these findings plasma-free serotonin levels are increased in breast cancer patients with advanced disease independently suggesting a role for 5-HT in breast tumor progression [58]. Similarly analyses of breast tumor gene expression profiles revealed that the transcript levels of several 5-HT receptors increased with tumor grade and metastatic propensity [53].

Expectedly, given the role of 5-HT in mouse mammary gland development, 5-HT increased the frequency of apoptotic cells in primary human mammary epithelial cells and in the immortal, but non-tumorigenic, breast epithelial MCF10A cell line; concurrently 5-HT decreased the frequency of proliferating cells [55]. By contrast, 5-HT did not affect the frequency of apoptotic cells in cultures of the MCF-7, MDA-MB-231 or T47D breast tumor cell lines [55]. Indeed 5-HT modestly stimulated the proliferation of MDA-MB-231 cells.

To learn whether the serotonergic system plays a functional role in human breast tumor cells survival or proliferation we assessed the activity of 5-HT antagonists in an independent study and found that they reduced the frequency of sphere forming cells in a panel of 8 breast tumor cell lines independent of their clinical or molecular subtypes (data not shown). The latter findings offer the prospect that some of these antagonists such as the SSRI may be repurposed to treat breast cancer.

It is also noteworthy that the serotonergic system has also been implicated in other malignancies including lymphoma and leukemia, prostate carcinomas, small cell lung carcinomas, glioblastomas, bladder carcinomas, colorectal carcinomas, hepatocellular carcinomas, cholangiocarcinomas, choriocarcinomas, carcinoid tumors and ovarian tumors (reviewed in [59]). Interestingly, overexpressing cDNAs encoding the 5-HT_2C_ or 5-HT_2A_ receptors results in the transformation of mouse 3T3 fibroblasts [60, 61].

We found that mouse mammary tumors arising in the MMTV-Neu N202 transgenic strain express TPH1, 5-HT and SERT in a high fraction of the cells comprising these tumors. Whereas we have not established the percentage of tumor cells expressing the 5-HT pathway components it seems that their frequency supersedes that of the BTIC fraction in tumors. Our immunofluorescence analyses of the expression of the latter serotonergic pathway components were unable to distinguish between cells that express high or low levels of these proteins. One possibility is that the extent to which the 5-HT pathway components are expressed differs between BTIC and their non-tumorigenic descendants. Additional experiments are required to determine whether both the tumorigenic and non-tumorigenic tumor cells in mammary tumors express the various 5-HT pathway proteins.

We focussed most of our studies on sertraline, which is a potent approved drug that selectively inhibits SERT activity. We found that sertraline irreversibly inhibited sphere-formation by BTIC-enriched tumor cells from independent mouse mammary tumors consistent with it targeting sphere-initiating cells. Sertraline also targeted BTIC *in vitro* as determined by tumor cell transplantation assays. Importantly, sertraline synergized with docetaxel to reduce sphere-formation and to shrink established tumors in mice. Each drug individually modestly reduced tumor-resident cell proliferation, but the combination of both drugs dramatically reduced tumor cell proliferation. Sertraline modestly increased the frequency of apoptotic tumor cells, but docetaxel had little effect on this mechanism of cell death. By contrast, the combination of both drugs markedly increased tumor cell apoptosis. Hence the drug combination reduced tumor cell proliferation and increased tumor cell apoptosis to a much greater extent than the effect of each drug alone consistent with them acting synergistically.

The residual tumors of those mice that had been treated with both sertraline and docetaxel comprised a reduced frequency of sphere forming cells compared to those of mice administered the vehicle suggesting that the drug combination reduced BTIC frequency in tumors. Our observations are consistent with the hypothesis that sertraline targets tumor-resident BTIC, whereas docetaxel targets primarily the non-tumorigenic cell compartment.

The biochemical mechanism(s) whereby 5-HT antagonists function to reduce BTIC activity are not known. One possibility is that the antagonists inhibit HER2 activity by reducing its tyrosine phosphorylation or by increasing its degradation. Whereas the latter is formally possible we found that human breast tumor cell lines that do not overexpress HER2 are as sensitive to 5-HT antagonists as those that do (data not shown). Hence the latter hypothesis seems unlikely. Efforts are underway to identify the signalling pathways downstream of SERT and 5-HT receptors that are required for BTIC activity.

Whether SSRI or other serotonergic system antagonists can be repurposed to treat breast cancer will depend in part on whether therapeutic concentrations can be achieved in patients. The concentration of sertraline in the plasma of individuals who were orally administered 200 mg of the drug is 0.19 ug/ml (0.55 μM), which was achieved between 4.5–8.4 hours post administration: the half-life of the drug is between 24 and 36 hours [62]. The plasma concentration of sertraline is directly proportional to the administered oral dose over the range of 20–400 mg. Moreover, daily oral doses of 400 mg of sertraline are well tolerated suggesting that a 1 μM plasma concentration, the approximate IC_50_ of the drug in breast tumor cell lines, can be achieved in humans [63]. The fact that sertraline synergizes with docetaxel when used at its IC_50_ in human breast tumor cell lines further suggests that therapeutic doses of this SSRI can be achieved in breast cancer patients.

In aggregate our data imply that 5-HT signalling is required to maintain BTIC activity and suggests that SSRI and other serotonergic system drugs might be repurposed to treat breast cancer patients in combination with chemotherapies or radiotherapy to achieve more durable breast cancer remissions than are attained currently. SSRI are among the most widely prescribed antidepressants, have been in use for decades, and are considered to be safe when used as prescribed [64].

It is noteworthy that epidemiologic studies have sought to determine whether SSRIs increase breast cancer recurrence, as a consequence of findings in experimental rodent models in the early 1990s suggesting that some antidepressants reduced the time of onset and increased the incidence of tumors (reviewed in [65]). However, later studies found that there is no association between SSRI therapies and breast cancer risk. For example, one recent study compared 2,129 women with primary invasive breast cancer who chronically took SSRI with 21,297 women selected at random. Women who took SSRI were not at increased risk of breast cancer than were those who did not take these antidepressants [66]. Another study of 1701 women with primary invasive breast cancer and 17,071 women selected randomly also did not find an association between short or long-term SSRI use and the risk of breast cancer [67]. The results of the latter studies suggest that SSRI like sertraline will not increase the incidence of breast cancer. To the best of our knowledge epidemiological studies have not addressed whether antidepressants reduce the risk of breast cancer, or whether their use during cytotoxic anticancer therapies improves patient survival or reduces breast cancer recurrence. In this regard we envision that any clinical trial of SSRI or other antidepressants as anticancer agents will occur by their use in combination with chemotherapy or radiotherapy during a finite treatment period.

## MATERIALS AND METHODS

### Care and treatment of mice

All procedures involving mice were performed with the approval of the Canadian Council on Animal Care.

### Tumor cell isolation and propagation

Mammary tumors were harvested from MMTV-Neu (N2O2 strain) transgenic mice [17] and processed to yield dispersed mammary tumor cells, which were placed in chemically-defined, serum-free medium containing B-27, Epidermal Growth Factor, Fibroblast Growth Factor 2 and heparin as previously described [6]. The dispersed tumorsphere-derived cells proliferate with a doubling time of roughly 12 hours during a 3–4 day period yielding spheres comprising between 400–800 cells. Tumorspheres arising during a 4-day period were dissociated by trituration and the dispersed cells seeded into fresh medium. The tumorspheres were serially passaged between 3–5 times before their use in the high-throughput screens and before assessing the activity of compounds in quantitative sphere-forming assays. Serial propagation of the tumorsphere-derived cells was limited to minimize the potential occurrence of genome alterations that might distinguish the sphere-resident cells from those comprising tumors.

### Small molecule high-throughput screen

To carry out the high-throughput screen tumorspheres were dissociated, the dispersed cells washed, and suspended into fresh medium containing the supplements identified above. The total cell number was determined using a hemocytometer: trypan blue (Gibco) was used to determine the frequency of non-viable cells, which typically was less than 5%. Cells were diluted to 20,000 cells/milliliter (mL) and 50 μL of the cell suspension pipetted into wells of a black 384-well plastic dish using a Beckman Coulter Biomek 3000 or Biomek FX. Following addition of cells and compounds in duplicate at a concentration of 5 μM, the plates were incubated for 24 hours at 37°C in a humidified 5% CO_2_ water-jacketed incubator. alamarBlue (5 μL) (Gibco) was then added to each well, and the plates are incubated for another 24 hours. Fluorescence was read at λ_excitation_ = 535 nm and λ_emission_ = 600 nm using a Perkin Elmer EnVision or a Beckman Coulter Multimode Detector Dx. High (+) control wells contained tumor cells in medium containing the vehicle; low (–) control wells contained the medium with the vehicle, but no cells.

### Sphere-forming assays

In preparation for sphere-forming assays, single cell suspensions comprising 6, 000 cells (in 200 μL) were dispensed into wells of a 96-well plate, and serial dilutions of the various compounds were then added into triplicate wells. Following a 4-day incubation period at 37°C, in a humidified atmosphere comprising 5% CO_2_, the spheres were counted and normalized to vehicle-treated control wells. Fresh compounds that were independently sourced from those used in the primary screen were obtained from Toronto Research Chemicals (Tocris).

### IC_50_ calculations

The 50% inhibitory concentration (IC_50_) of compounds was calculated using GraphPad Prism 6 software. X-axis were X = Log(X) transformed and then fit with a dose-response curve. The DMSO vehicle control was included to aid IC_50_ calculations and was assigned a 1nanomolar concentration of the tested compound.

### Animal studies

Female FVB/N mice were used as recipients for transplanting primary tumor cells or tumorsphere-derived cells isolated from the MMTV-Neu (N202) transgenic strain. Single cell suspensions were prepared at a 2X concentration in phosphate buffered saline (PBS)/5% fetal bovine serum prior to being mixed with Matrigel™ (BD Biosciences) in a 1:1 ratio as described previously [6, 29, 31]. One hundred microliters of the resulting tumor cell suspensions were injected subcutaneously into the fat pad of the #2 mammary glands of the recipient mice. More often than not, all the mice developed palpable tumors whose volumes were measured using a caliper. Drug treatments were initiated when tumors reached a volume of ~700 mm^3^. The tumor-bearing mice were treated with sertraline (60 mg/kg) [Tocris], docetaxel (10 mg/kg) [LC Labs), both sertraline and docetaxel, or an equivalent volume of the vehicle by intraperitoneal injection. Thereafter tumor volume was measured once weekly. The mice were harvested at end-point, when the volume of the tumor comprised ~10% the weight of the mouse.

### Histology, immunofluorescense and immunohistochemical analyses

Tumor fragments were fixed in 4% paraformaldehyde, embedded in paraffin, sectioned at 5 microns, and subsequently de-paraffinized and rehydrated in ethanol (100%–70% gradient). The tumor sections were stained with hematoxylin and eosin to reveal their histology. Antigen retrieval, immunofluorescence and immunohistochemical analyses of the tumor sections were performed as previously described [6, 29, 31, 68, 69]. A polyclonal antibody to SERT (Alomone Labs; AMT-004), elicited by immunization of rabbits with a peptide corresponding to amino acids 388-400 in the fourth extracellular loop, was used to detect the protein in mouse mammary tumor sections. The antibody to SERT was diluted 1:100 and the antigen antibody complexes were visualized after incubation with AlexaFluor 594 coupled to a goat anti rabbit secondary antibody, which was diluted 1:200 before use. A polyclonal antibody to TPH1 (LifeSpan BioSciences, Inc.; LS-C117936) generated by immunizing rabbits with a synthetic peptide (amino acids 231-280) was used to detect the protein in sections of mammary tumors. The antibody to TPH1 was diluted 1:100 and the antigen antibody complexes were visualized with AlexaFluor 594 coupled to a goat anti rabbit secondary antibody, which was diluted 1:200 before use. A mouse monoclonal antibody to 5-HT (Novus Biologicals; 5HT-H209) was used to detect the neurotransmitter in tumor sections. The antibody to 5-HT was diluted 1:20 before use and the antigen antibody complexes were identified with an AlexaFluor 488 coupled goat anti mouse antibody diluted 1:200 before use. An antibody to Ki67 (ABCAM, Cambridge, Massachusets) was used to identify proliferating tumor cells, and TUNEL assays were performed on tumor sections to identify apoptotic cells as described previously [29, 31].

### Combination matrix models

Synergy was calculated using the Bliss independence model where *I_A_* and *I_B_* indicate the % inhibition of cell viability achieved by either single agent, and *I_Bliss-Independence_* indicates the predicted % inhibition of viability.

$$I_{Bliss-Independence}=I_{A}+I_{B}-(I_{A}\;I_{B})$$

### Statistical analyses

Assays were repeated in 3 biological experiments with each data point being the average of 3 technical replicates within each biological experiment. Where relevant the Figures show the mean +/− the standard deviation. Differences among experimental means were analyzed by analysis of variance (one-way ANOVA) using Graphpad Prism 6 (La Jolla, CA). Significant differences between individual means were calculated using Tukey's test. For Kaplan-Meier survival, significance was determined using a log-rank (Mantel-Cox) test. Differences were considered statistically significant if *P* < 0.05.

## SUPPLEMENTARY MATERIALS FIGURES AND TABLE


