# Supplementary material for: Serotonin transporter antagonists target tumor-initiating cells in a transgenic mouse model of breast cancer
Source: Oncotarget. 2016 Jul 15;7(33):53137–52. doi: 10.18632/oncotarget.10614 (PMC5288174; doi:10.18632/oncotarget.10614)
Supplement: Supplementary file 1 [file oncotarget-07-53137-s001.pdf]

Serotonin transporter antagonists target tumor-initiating cells in a transgenic mouse model of breast cancer

Supplementary Materials

A

	Number of Wells with Single Cells	Number of Wells with a Single Sphere	Frequency of Wells with Spheres (%)
15 96-well plates	601	11	1.8
16 96-well plates	336	3	0.9
16 96-well plates	447	15	3.2
			**Mean** = 2.0

B

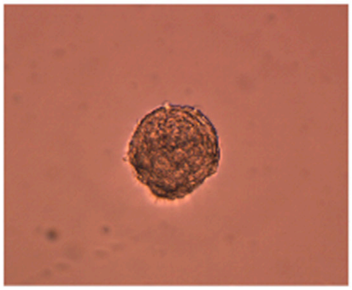

Supplementary Figure S1: Single mouse mammary tumorsphere-derived cells form spheres. (**A**) Single mouse mammary tumor cells from 3 independent mouse mammary tumors were seeded into the wells of 15 or 16 96-well plates and the number of wells with single cells identified a day later; wells seeded with tumorspheres were enumerated a week after the single cells were seeded. (**B**) Image of a well comprising a single tumorsphere.

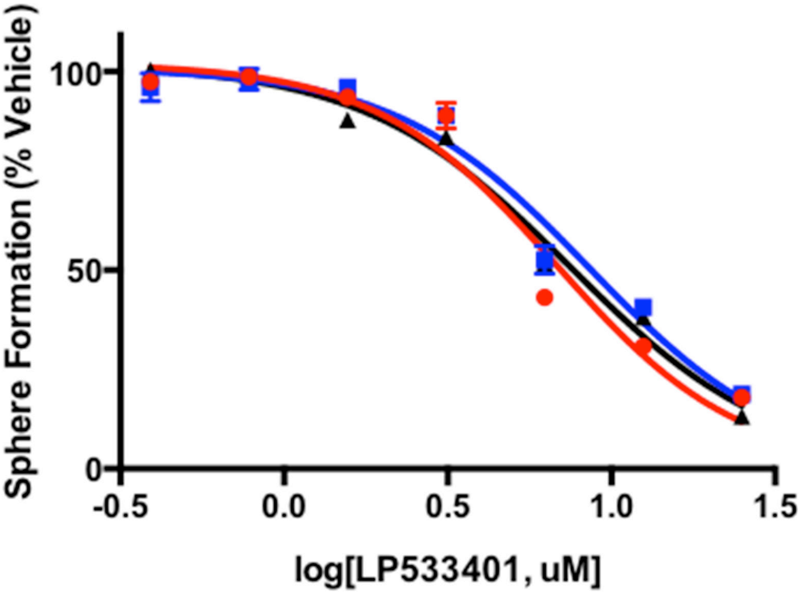

Supplementary Figure S2: An inhibitor of TPH1 reduces sphere formation in a dose-dependent fashion. Dilutions of LP533401were assayed in triplicate replicates for its capacity to affect sphere formation by tumorsphere-derived cells isolated from 3 independent tumors.

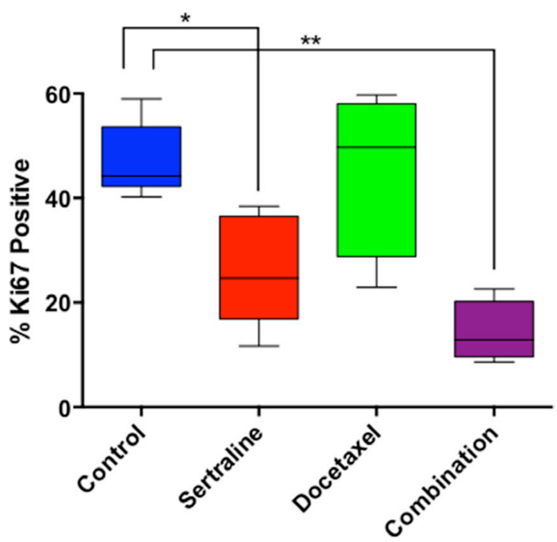

Supplementary Figure S3: Sertraline and docetaxel synergize to reduce the fraction of Ki67-positive cells. *P*-values were calculated using one-way ANOVA; (*) < 0.05; (**) < 0.01. $P = 0.0009$.

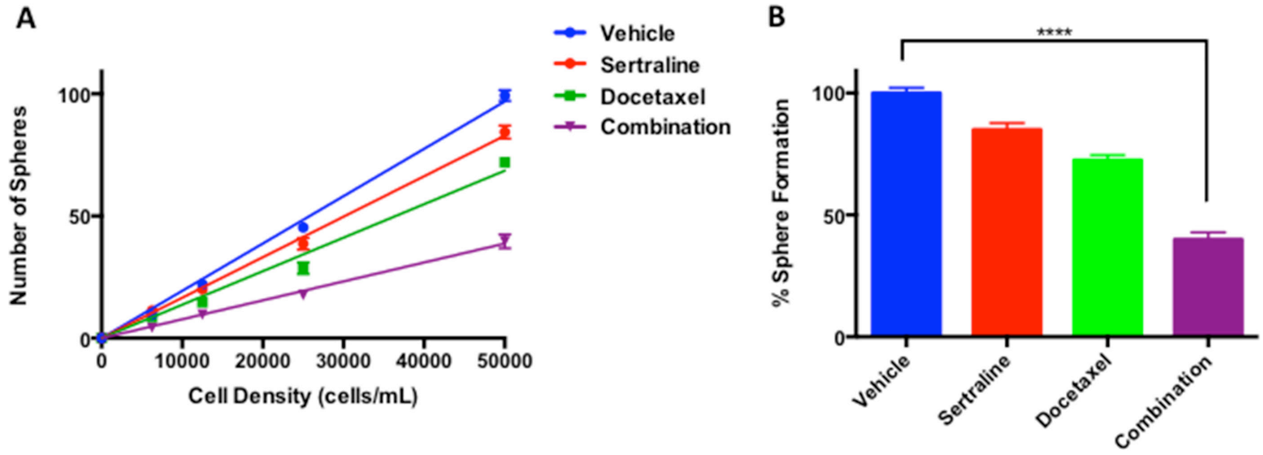

Supplementary Figure S4: Limiting dilution tumorsphere-forming assays reveal that tumors harvested from mice administered sertraline and docetaxel comprised fewer sphere initiating cells relative to the other treatment groups. (**A** and **B**) Frequency of tumorsphere forming cells in residual tumors remaining after treating tumor-bearing mice with the vehicle, sertraline, docetaxel, or the combination of sertraline and docetaxel. *P*-values were calculated using one-way ANOVA; (****) < 0.00001.

Supplementary Table S1: Selective antagonists of 4 different 5-HT receptors reduced the frequency of tumorsphere-forming cells

Selective Antagonist	5-HT Receptor Target	IC50 Prestoblue Reduction	IC50 Sphere Formation
SB 2242489	1B	2.8 µM	0.6 µM
SB 216641	1B	3.4 µM	3.3 µM
N -Desmethylclozapine	2C	7.0 µM	9.0 µM
SB 699551	5A	0.3 µM	0.5 µM
MS 245	6	5.1 µM	6.1 µM

The IC_{50} of selective 5-HT receptor antagonists was determined in sphere forming assays in triplicate at each concentration of the inhibitor.
